# Genome-Wide Identification and Expression Analysis of the Dof Transcription Factor Family in *Prunella vulgaris*

**DOI:** 10.3390/ijms27031354

**Published:** 2026-01-29

**Authors:** Ming Zhang, Yong Wu, Lei Xu, Ru Xu, Yutao Yao, Lichun Ye, Zhaohua Shi

**Affiliations:** 1 School of Pharmacy, Hubei University of Chinese Medicine, Wuhan 430065, Chinawuhanxulei@163.com (L.X.);; 2 Hubei Shizhen Laboratory, Hubei University of Chinese Medicine, Wuhan 430065, China; 3 School of Traditional Chinese Medicine, Hubei University of Chinese Medicine, Wuhan 430065, China; 4 Ministry of Education Key Research Project on Chinese Medicinal Resources and Compound Formulas, Hubei University of Chinese Medicine, Wuhan 430065, China

**Keywords:** *Prunella vulgaris*, Dof transcription factor, gene family, biosynthesis regulation, rosmarinic acid

## Abstract

The Dof (DNA binding with one finger) transcription factor family is a plant-specific group of transcription factors that play critical roles in plant growth and development, stress response, and the regulation of secondary metabolism. *Prunella vulgaris* (*P. vulgaris*) has attracted considerable attention due to its medicinal value, with rosmarinic acid being one of its key bioactive components. However, the systematic identification of the Dof transcription factor family in *P. vulgaris* and its regulatory role in rosmarinic acid biosynthesis remains poorly understood. In this study, based on the whole-genome data of *P. vulgaris*, we identified 48 Dof transcription factor genes distributed across 14 chromosomes using bioinformatics approaches. Physicochemical analysis revealed that the encoded proteins have molecular weights ranging from 15,482.44 to 55,875.53 Da, amino acid lengths between 142 and 509, and theoretical isoelectric points from 4.84 to 10.2. All proteins were predicted to be hydrophilic and localized in the nucleus. Phylogenetic analysis classified them into four subfamilies, and multiple sequence alignment confirmed that all members contain a conserved C2-C2-type zinc finger domain. Analysis of cis-regulatory elements in the promoter regions identified numerous elements related to light responsiveness, hormone response, and development. Transcriptomic expression profiling demonstrated distinct tissue-specific expression patterns of Dof genes, with some showing high expression in spikes and seeds. Correlation analysis between gene expression and rosmarinic acid content identified three candidate genes potentially involved in the regulation of rosmarinic acid biosynthesis, which were further validated by RT-qPCR. Moreover, protein–protein interaction network predictions indicated 242 interactions among 23 Dof proteins. This study provides the first systematic identification of the Dof transcription factor family in *P. vulgaris*, offering important insights into the transcriptional regulation of rosmarinic acid biosynthesis and presenting potential genetic targets for enhancing rosmarinic acid production through genetic engineering.

## 1. Introduction

*Prunella vulgaris* L. (commonly abbreviated as *P. vulgaris*), a perennial herb of the family Lamiaceae, is an important traditional Chinese medicine. Its dried fruit spike is the official medicinal part. First documented in the *Compendium of Materia Medica* (Ben Cao Gang Mu), it is reported to have pharmacological properties such as clearing heat-toxicity, reducing swelling, and dissipating nodules. The plant’s status and efficacy are formally recognized by its consistent inclusion as an official medicinal material in all successive editions of the *Chinese Pharmacopoeia*, which underpins its widespread clinical application [1,2]. *P. vulgaris* contains various secondary metabolites such as phenolic acids and triterpenoids, including common compounds like caffeic acid, rosmarinic acid, ursolic acid, and oleanolic acid. These compounds exhibit pharmacological activities such as anti-inflammatory, antimicrobial, immunomodulatory, and hepatoprotective effects [3,4,5]. To elucidate the biosynthesis of active ingredients in *P. vulgaris* and promote the comprehensive development and application of its plant resources, in-depth research on its biosynthetic regulatory mechanisms is necessary. This will not only help enhance the medicinal value of *P. vulgaris* but also provide a more solid scientific basis for its clinical application.

Rosmarinic acid is a quality control marker component for *P. vulgaris*. It is widely distributed in the Nepetoideae subfamily of Lamiaceae. In plants, its biosynthesis primarily proceeds via the phenylpropanoid pathway: phenylalanine is converted to cinnamic acid by phenylalanine ammonia-lyase (PAL) [6,7]. Through a series of enzymatic reactions, cinnamic acid is transformed into p-coumaric acid and then caffeic acid. Caffeic acid is further converted to rosmarinic acid via enzymes, including caffeic acid O-methyltransferase, leading to the formation of rosmarinic acid from p-coumaric acid and 3,4-dihydroxyphenyllactic acid. Currently, the biosynthetic pathway of rosmarinic acid in *P. vulgaris* has been identified through transcriptomic and proteomic studies [8,9]. However, reports on how transcription factors (TFs) regulate the synthesis of secondary metabolites in *P. vulgaris* are scarce, which severely hinders the understanding of secondary metabolite biosynthesis in this plant. However, the transcriptional regulatory network controlling RA biosynthesis in *P. vulgaris*, especially the roles of specific TF families, remains a critical knowledge gap. While MYB and bHLH proteins are established master regulators of phenylpropanoid metabolism [10], emerging models position Dof TFs as essential upstream modulators or co-regulators. They can integrate developmental and environmental signals to fine-tune the pathway, either by directly binding to biosynthetic gene promoters or, as recently elucidated, by forming protein–protein interaction complexes with MYB factors to modulate their activity [11]. Given the established role of Dof TFs in regulating phenolic acid biosynthesis in the closely related Lamiaceae species S. miltiorrhiza [12,13], and the shared RA biosynthetic pathway, we hypothesize that Dof family members perform a similarly pivotal and specific regulatory function in *P. vulgaris*. Therefore, a systematic identification and characterization of the Dof TF family in *P. vulgaris* constitutes a necessary and rational foundational step toward elucidating the transcriptional control of its valuable medicinal compounds. Based on genomic data constructed using high-throughput sequencing methods, this study systematically analyzes the Dof TFs in *P. vulgaris* using bioinformatics approaches, laying the foundation for further investigation into the functions of the Dof TF family genes in *P. vulgaris*.

Transcription factors are protein molecules that can specifically bind to upstream regulatory sequences of genes. Through their interactions with these sequences and with other related proteins, they activate or inhibit transcription, thereby ensuring the expression of target genes at specific strengths, times, and locations. This mechanism forms a complex regulatory network that finely modulates plant secondary metabolism [14,15]. The Dof (DNA binding with one finger) family is a plant-specific TF family belonging to the single zinc finger superfamily. It plays important roles in plant growth and development, stress response, and the regulation of secondary metabolism. The conserved Dof domain typically consists of 200 to 400 amino acids, featuring a conserved DNA-binding domain at the N-terminus and a non-conserved transcriptional regulation region at the C-terminus [16]. The DNA-binding domain is a C2-C2-type zinc finger domain comprising 50 to 52 amino acid residues, including four absolutely conserved cysteine residues that covalently coordinate a Zn^2+^ ion [17,18].

The Dof TF family plays significant roles in various aspects of plant growth and development, stress responses, and metabolic regulation. Dof TFs were first discovered in maize (*Zea mays*) in 1993, where they were found to participate in the transcriptional regulation of genes related to light response and carbon metabolism [19]. The binding site recognized by the C2-C2 zinc finger domain of Dof TFs is typically the AAAG sequence, although exceptions exist; for instance, the AOBP Dof TF in pumpkin (*Cucurbita moschata*) specifically recognizes the AGTA sequence [20]. This C2-C2 zinc finger domain is not only the characteristic conserved domain of Dof TFs but also controls the binding of Dof TFs to target genes and influences their interactions with other TFs. Studies have shown that the OBP1 Dof TF in *Arabidopsis thaliana* can interact with bZIP TFs through its conserved domain, thereby enhancing the binding of bZIP TFs to target genes [21]. Additionally, the SAD Dof TF in barley (*Hordeum vulgare*) interacts with the GAMYB MYB TF [22]. These findings indicate that the conserved Dof domain can interact with various TFs, such as WRKY, bZIP, and MYB proteins, thereby influencing gene expression at different levels. These interactions are involved in multiple biological processes, including plant growth and development, stress resistance responses, fruit development, storage substance accumulation, and the transcriptional regulation of certain secondary metabolites [23].

Importantly, converging evidence highlights the direct involvement of Dof TFs in regulating the phenylpropanoid pathway and its branches leading to phenolic acid biosynthesis. For instance, in *Arabidopsis thaliana*, the Dof protein OBP3 has been shown to physically interact with MYB transcription factors to co-regulate the expression of genes encoding key enzymes like PHENYLALANINE AMMONIA-LYASE (PAL) and 4-COUMARATE:CoA LIGASE (4CL), thereby influencing lignin and flavonoid production [12]. More directly relevant to rosmarinic acid (RA) biosynthesis, studies in the medicinal Lamiaceae species *Salvia miltiorrhiza* (Danshen) have provided important clues. Genome-wide analysis of the Dof family in S. *miltiorrhiza* has revealed that the expression profiles of several *SmDof* genes are closely correlated with the accumulation of phenolic acids, including salvianolic acids, strongly suggesting their potential regulatory roles in the phenylpropanoid pathway [24]. Furthermore, key enzyme genes in the RA biosynthetic pathway of S. *miltiorrhiza*, such as rosmarinic acid synthase (*SmRAS*), have been shown to be under tight transcriptional control [25,26].Although direct functional evidence linking specific Dof TFs to RA pathway genes in *P. vulgaris* is currently lacking, the established role of Dofs in regulating phenolic acid metabolism in this closely related species provides a compelling rationale for our hypothesis. It strongly suggests that Dof family members are likely to perform specific regulatory functions in the RA biosynthesis of *P. vulgaris*.

## 2. Results

### 2.1. Identification of Dof Family Genes, Analysis of Physical and Chemical Properties, and Chromosomal Localization of P. vulgaris

Through comparative analysis using the HMM library and BLASTP (https://blast.ncbi.nlm.nih.gov/Blast.cgi, accessed on 16 June 2025) alignment, 48 Dof gene family members were identified. In *P. vulgaris*, the relative molecular masses of Dof family proteins ranged from 15,482.44 to 55,875.53. The amino acid count varied significantly, with PvDof13 containing the most amino acids (509) and PvDof32 the least (142). Their isoelectric points ranged from 4.47 (PvDof43) to 9.97 (PvDof20). Subcellular localization predictions indicated that all 48 Dof proteins were localized in the nucleus. The hydrophilicity 2 index ranges from −0.353 to −0.978, indicating all proteins are hydrophilic. Analysis of the instability index reveals that, except for PvDof5, which belongs to stable proteins, the rest are unstable proteins, potentially related to plant stress resistance. Through secondary structure prediction of Dof family members in *P. vulgaris*, the results show that all members’ protein peptide chains consist of four typical structural elements: α-helices, extended chains, β-turns, and random coils. However, the proportions of these four structures exhibit significant differences. Overall, α-helices and random coils dominate as the primary structural components of proteins. The proportion of α-helix structures ranges from 4.68% (PvDof29) to 32.01% (PvDof14), while random coil structures account for 45.45% (PvDof39) to 72. 18% (PvDof33). β-sheet structures have the smallest proportion, ranging from 3. 14% (PvDof12)to 12.50% (PvDof44). In terms of quantity and proportion, the fourth element, random coil, was the main component of all 48 members, while the first element, α-helix, and the second element, extended chain, were the secondary components (Supplementary Table S1).

**Figure 1:**
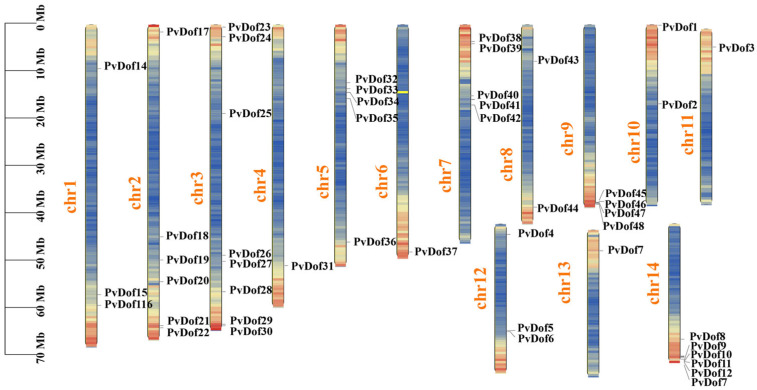
Chromosomal location distribution of Dof genes in *P. vulgaris*.

Chromosomal distribution analysis of the 48 identified Dof genes showed uneven distribution across 14 chromosomes in *P. vulgaris*. Chromosome 3 contained the highest number of Dof genes (8), followed by chromosomes 2 and 14 (6 each). Chromosomes 5 and 7 each had five Dof genes, chromosome 9 had four, chromosome 12 had three, chromosomes 8 and 10 each had two, while chromosomes 4, 6, 11, and 13 each contained only one Dof gene (Figure 1).

### 2.2. Phylogenetic Analysis and Collinearity Analysis of Dof Gene Family in P. vulgaris

**Figure 2:**
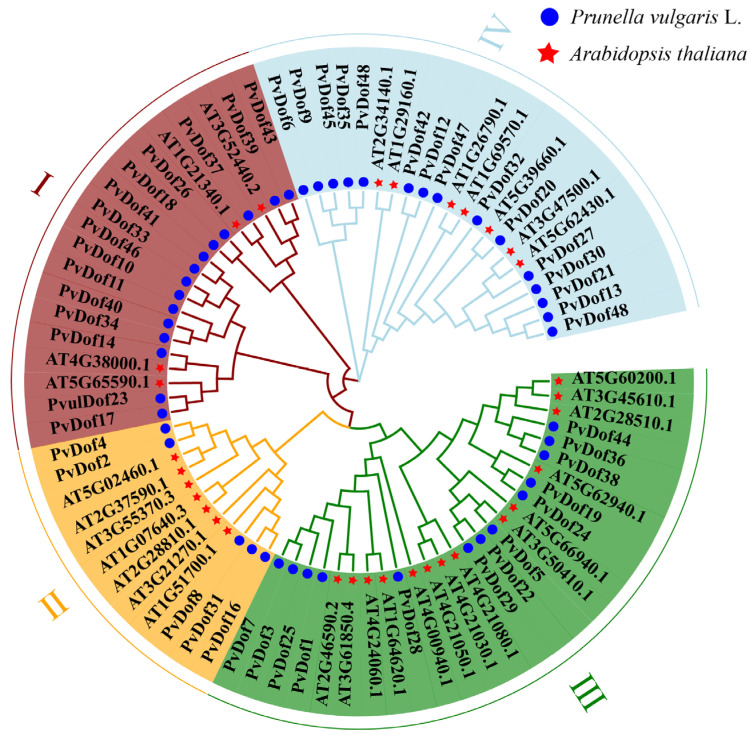
Phylogenetic analysis of Dof family members in *P. vulgaris* and *Arabidopsis thaliana*.

The Dof family proteins in *P. vulgaris* were classified according to the classification method of Dof proteins in *Arabidopsis thaliana*. The Dof transcription factors in *P. vulgaris* were divided into four subgroups. Among them, subgroup I and subgroup IV contained the most Dof members, with 15 Dof members, respectively. Subgroup III contained 13 Dof transcription factors, which was the second largest subgroup. Subgroup II contained the fewest Dof members, with five Dof members (Figure 2).

**Figure 3:**
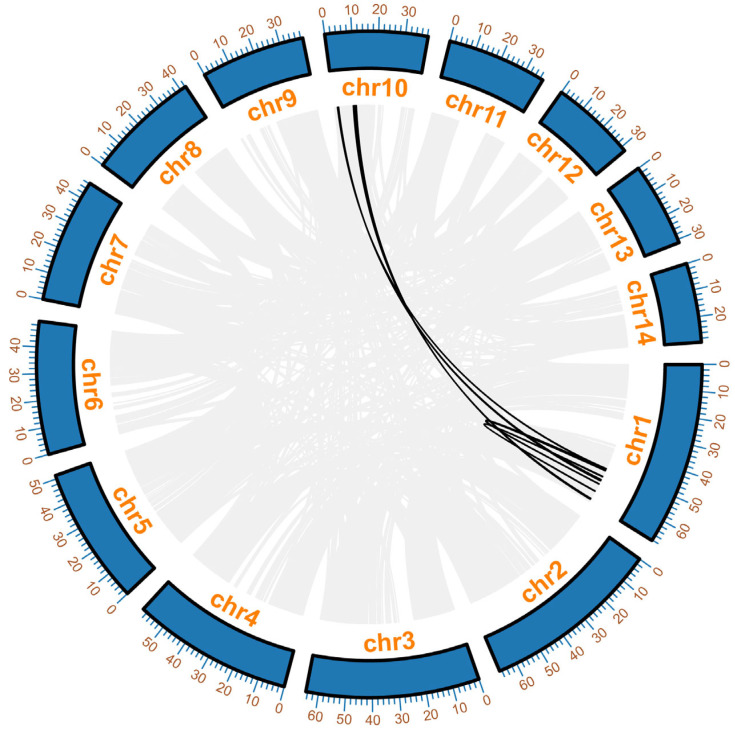
Analysis of the whole-genome replication of Dof gene in *P. vulgaris*.

To investigate the evolutionary relationships among Dof family members in the *P. vulgaris* genome, we analyzed the genome-wide duplication events of its Dof genes (Figure 3). Gray lines represent all duplicated gene pairs within the *P. vulgaris* genome, while black lines denote the duplicated gene pairs in the Dof gene group. Co-linearity analysis revealed shared co-linearity patterns between chromosomes chr1 and chr 10. Specifically, three duplicated gene pairs were identified between chr10 and chr1, with three additional pairs present within chr1. These findings suggest that these genes may have originated from single or multiple duplication events, with genome-wide duplication events likely being a key driver of the evolutionary divergence in the Dof gene family.

**Figure 4:**
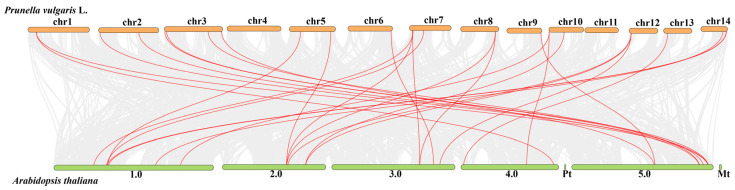
Synteny analysis of Dof genes in *P. vulgaris* and *Arabidopsis thaliana*.

The analysis of collinearity between 14 chromosomes in *P. vulgaris* and 5 chromosomes in *Arabidopsis thaliana* revealed that gray lines represent all collinear gene pairs between the two species, while red lines indicate Dof gene collinearity (Figure 4). Results showed that all chromosomes in *P. vulgaris* except chr4 and chr11 contain collinear gene pairs with *Arabidopsis thaliana*. Specifically, 27 Dof family members in *P. vulgaris* formed collinear gene pairs with *Arabidopsis thaliana* Dof members. Chromosomes chr3 and chr7 exhibited the highest collinearity with 4 pairs each, whereas chr6, chr9, and chr13 each had only one collinear pair.

### 2.3. Analysis of the Conservative Domain and Gene Structure of Dof Family Members in P. vulgaris

**Figure 5:**
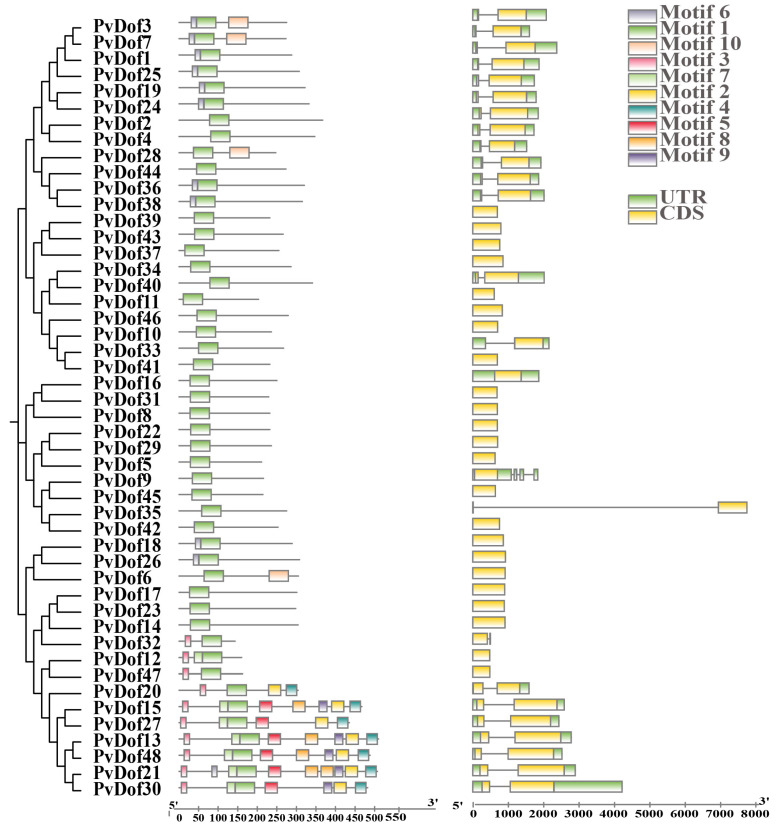
Visualization of the Dof gene structure in *P. vulgaris*.

To investigate the potential functions of Dof gene family members in *P. vulgaris*, we performed motif analysis using the MEME website. The analysis identified 10 conserved motifs (Supplementary Table S2). Structural domain analysis of all Dof members in *P. vulgaris* (Figure 5) revealed that motif 1 is present in all members, indicating it as a characteristic domain. Other motifs are exclusive to specific subfamilies. The conservation and arrangement of motifs among Dof members within the same subfamily show similarity, suggesting analogous functions among members of the same subfamily. This highlights both structural similarity and diversity in *P. vulgaris* Dof proteins. Notably, motifs 3, 4, 5, and 9 are exclusively found in Group IV.

**Figure 6:**
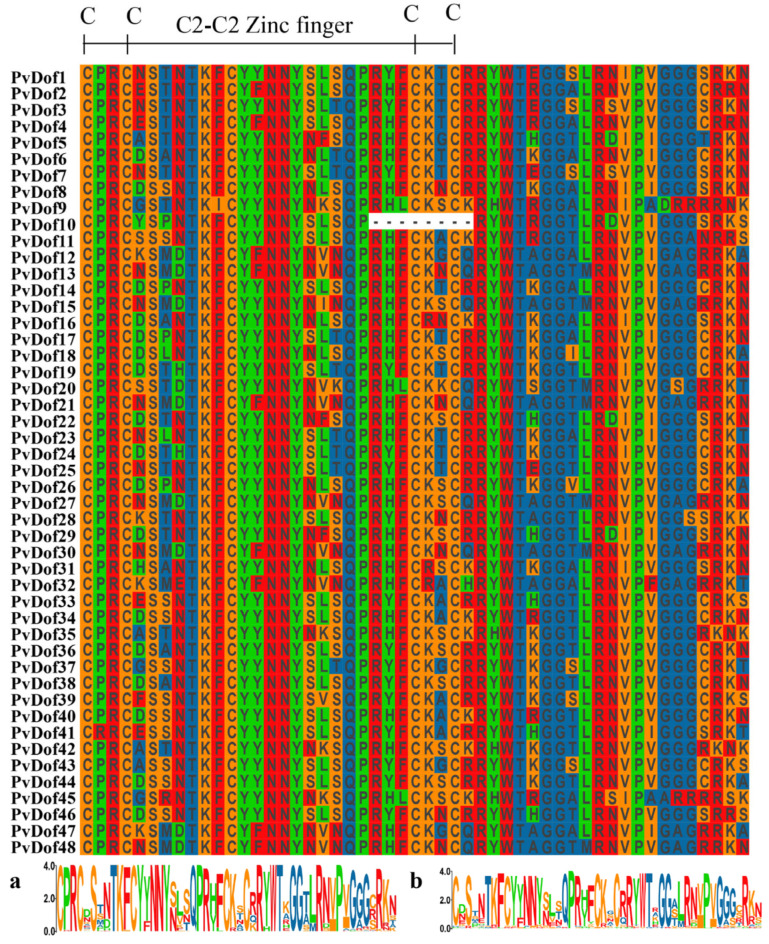
Multisequence alignment of Dof members in *P. vulgaris*; functional domains of *Arabidopsis* (**a**) and *P. vulgaris* Dof (**b**).

Genetic structure analysis revealed that the Dof member genes of *P. vulgaris* exhibit fewer introns, with closely related genes displaying similar intron-exon structures. To better characterize the Dof domain, we performed multi-sequence alignment using Genedoc (version 2.7) software (Figure 6). The motif 1 sequence of Dof proteins is highly conserved, featuring a distinct C-C-C-C domain and the conserved motifs TKFCY, NNY, and QPR. The motif 1 domain of Dof proteins also shows structural similarity to the Dof domain sequence model, further confirming the identified protein.

### 2.4. Analysis of Cis-Regulatory Elements of Dof Gene in P. vulgaris

**Figure 7:**
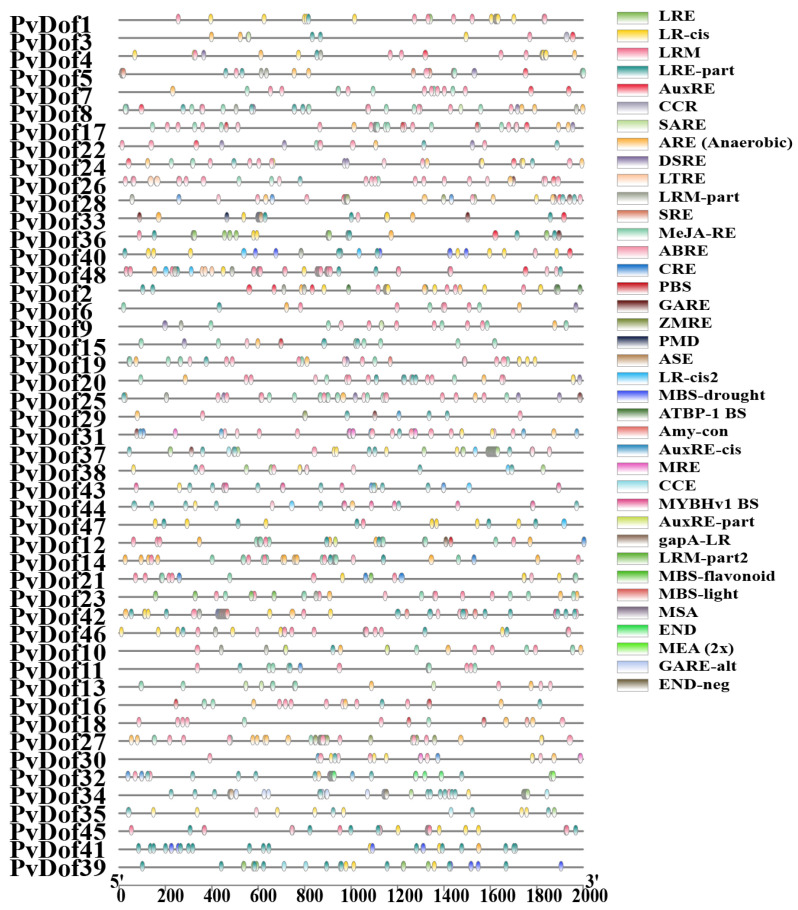
Analysis of cis-regulatory elements of Dof gene family members in *P. vulgaris*.

**Figure 8:**
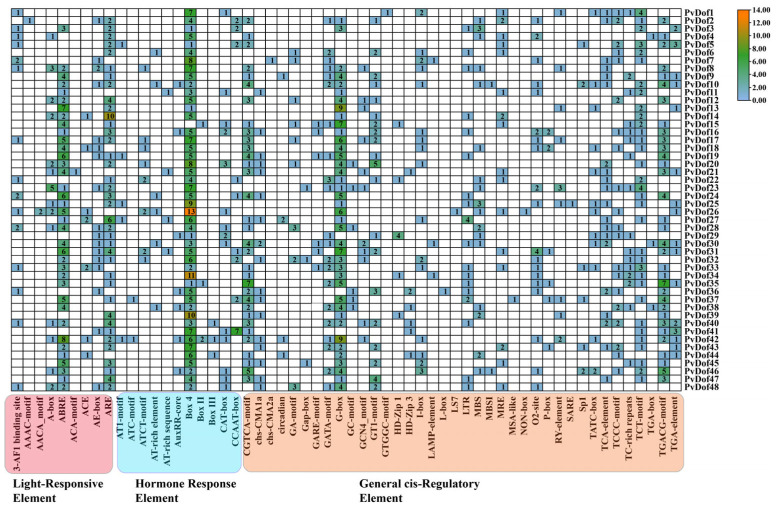
Heatmap visualization of cis-acting elements.

To investigate the diverse functions of the *P. vulgaris* Dof gene family, we analyzed 2000 base pairs of upstream promoter regions from 48 Dof genes using the PlantCARE database (Figure 7 and Figure 8). The study identified multiple cis-acting elements involved in plant development, cell cycle regulation, seed-specific expression, and tissue-specific expression (e.g., endosperm and meristem). These elements encompassed light-responsive elements, hormone-responsive elements, and stress-related elements (e.g., AREs). Among them, ABREs (abscisic acid-responsive elements) are primarily involved in abscisic acid (ABA) response. Hormone-responsive elements were predominant, with the CGTCA-motif and TGACG-motif (associated with methyl jasmonate/MeJA response) being notably abundant. General transcriptional regulatory elements participate in fundamental transcriptional regulation by binding to various transcription factors, thereby influencing gene expression under different conditions. For instance, chs-CMA1a and chs-CMA2a (cis-acting elements of chalcone synthase genes) are involved in regulating flavonoid biosynthesis. Circadian (diurnal) rhythms may also contribute to the regulation of plant circadian cycles. Overall, among the Dof transcription factor family in *P. vulgaris*, BOX elements are present and highly expressed in nearly all Dof family members. Elements such as 4ABRE, ARE, BOX4, CGTCA-motif, G-BOX, TCT-motif, and TCGCT-motif are present in most Dof family members. Additionally, motifs like AAC-motif, AACA-motif, ACA-motif, chs-CMA2a, GTGGC-motif, L-BOX, LS7, MSA-like, and NON-box are also present. SAREs are expressed in specific genes. The detailed names and descriptions of all cis-acting elements (Supplementary Table S3).

### 2.5. Analysis of Protein Interaction Network of Dof Gene Family Members in P. vulgaris

**Figure 9:**
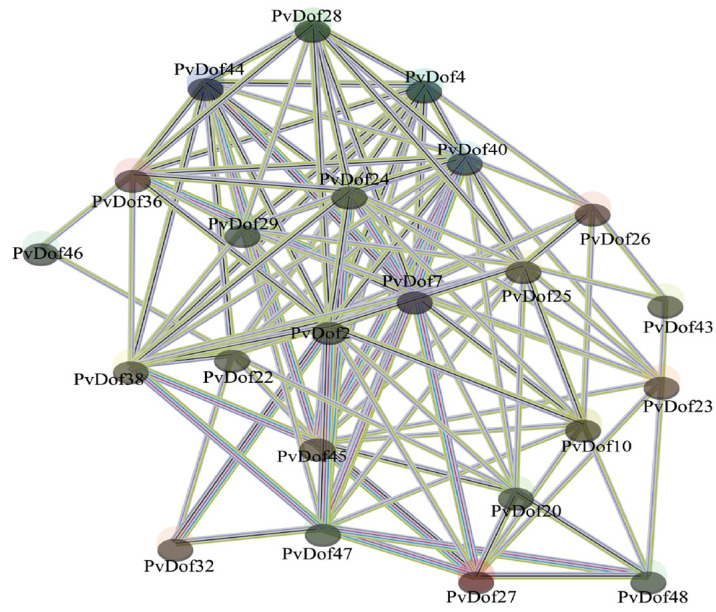
Analysis of Dof protein interaction prediction in *P. vulgaris*.

By investigating the potential functions of *P. vulgaris* Dof proteins and their interactions with family members, the following results were obtained (Figure 9): The regulatory network contained 23 nodes (representing Dof proteins) and 242 edges (representing protein–protein interactions), indicating diverse interaction patterns among *P. vulgaris* Dof proteins. The potential interactions among the 23 Dof members provide a crucial foundation for further validation of the transcriptional regulation by *P. vulgaris* Dof transcription factors.

### 2.6. Screening of Candidate Dof Gene for Regulation of Rosemary Acid Biosynthesis in P. vulgaris

The preliminary analysis using SPSS (version 28.0.1.1) software identified three candidate genes potentially involved in regulating the biosynthesis of rosmarinic acid, with their specific correlations as shown in the table below (Table 1).

### 2.7. Analysis of the Expression Pattern of Dof Gene Family in P. vulgaris

**Figure 10:**
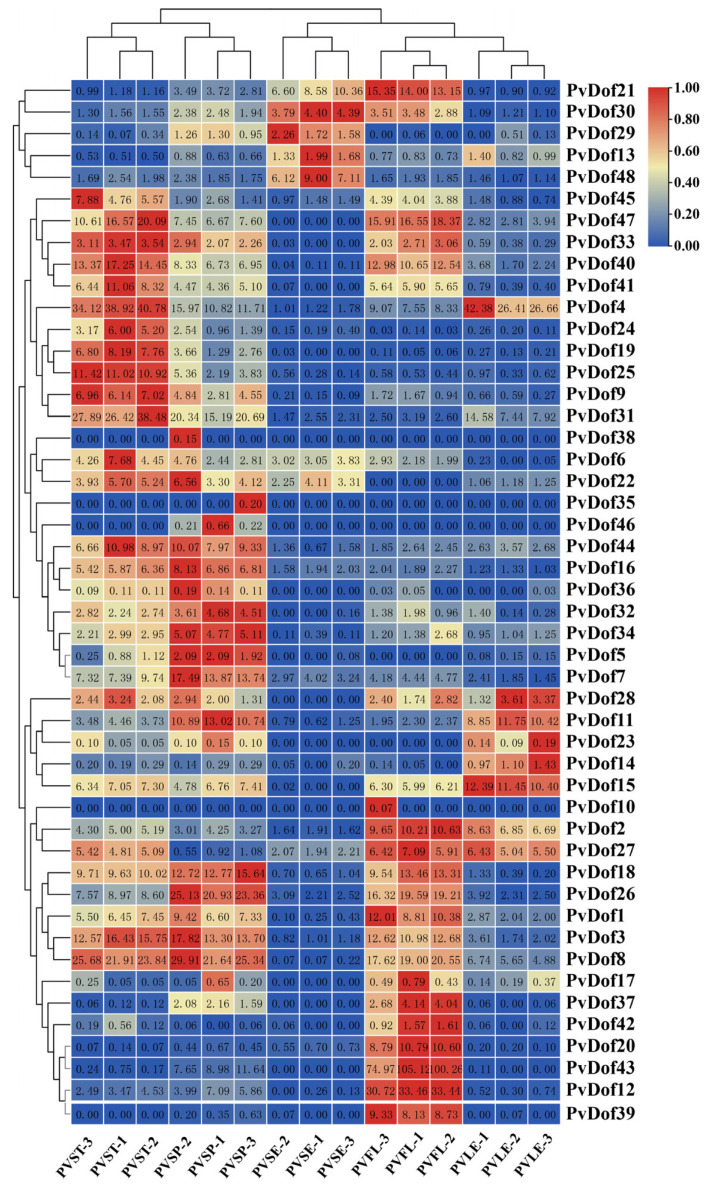
Analysis of protein expression pattern of Dof gene family members in *P. vulgaris*.

To investigate the potential role of Dof gene in the growth and development of *P. vulgaris*, the expression patterns of Dof gene in five different tissue locations were analyzed using the transcriptomic data obtained by our research team (Figure 10).

**Figure 11:**
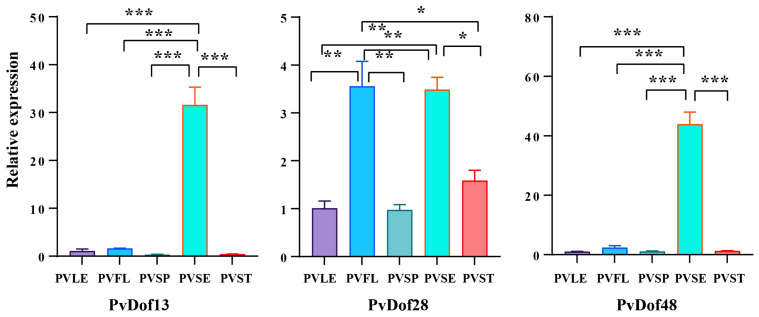
qPCR gene expression analysis. * *p* < 0.05, ** *p* < 0.01, * *p* < 0.001.

The results revealed distinct tissue-specific expression patterns among Dof transcription factors, demonstrating remarkable diversity. Most members were expressed across all tissues, with the following notable exceptions: *PvDof38*, *PvDof35*, *PvDof46*, *PvDof23*, *PvDof10*, and *PvDof17* showing low or no expression. *PvDof21*, *PvDof47*, *PvDof40*, PvDof26, *PvDof20*, *PvDof43*, and *PvDof12* exhibited high expression in floral tissues, suggesting potential involvement in regulating *P. vulgaris* flowering. *PvDof4* was highly expressed in leaves and stems, while PvDof11 showed elevated expression in both leaves and panicles. *PvDof19*, *PvDof 25*, and *PvDof31* demonstrated significantly higher stem concentrations compared to other tissues, with *PvDof31* additionally showing high expression in stems and panicles. We further examined the expression patterns of candidate genes potentially associated with rosmarinic acid accumulation. *PvDof13* and *PvDof48* displayed seed-specific high expression, a pattern evident in the transcriptome data. In contrast, *PvDof28* exhibited a more complex expression profile. Although transcriptomic data indicated higher expression in flowers and spikes with minimal signal in seeds, subsequent qPCR validation demonstrated that *PvDof28* was expressed across all tissues, with the highest transcript levels in seeds and flowers. The discrepancy regarding its expression in seeds likely stems from its relatively low transcript abundance, which falls near the detection limit of RNA-seq, potentially leading to underestimation. The greater sensitivity of qPCR provides a more reliable quantification of its expression dynamics. Collectively, qPCR results confirmed the seed-specific expression of *PvDof13* and *PvDof48* and clarified the true expression pattern of *PvDof28* (Figure 11).

Given that the fruit spike is the primary medicinal part of *P. vulgaris*, genes with high expression in spikes or seeds—such as *PvDof13*, *PvDof48*, and *PvDof28*—may be involved in regulating the biosynthesis of specialized metabolites, including rosmarinic acid, and thus warrant further functional investigation.

## 3. Discussion

Dof transcription factors play crucial roles in plant growth, development, and the biosynthesis of secondary metabolites. Studies have demonstrated their ability to specifically regulate multiple secondary metabolic pathways. Using the medicinal plant *P. vulgaris* as a model, this study systematically identified Dof transcription factor gene family members involved in rosmarinic acid synthesis. Through homology sequence alignment and physicochemical property analysis, we observed significant variations in sequence length and chemical characteristics among *P. vulgaris* Dof family members. Despite these differences, all members conservatively contain C2-C2-type single zinc finger domains and are predominantly localized in the nucleus, indicating their fundamental function in transcriptional regulation. This coexistence of sequence diversity and structural conservation likely reflects plants’ adaptive mechanisms through functional specialization to achieve precise control over diverse biological processes in complex environments. The phylogenetic classification of these genes into four subgroups, consistent with patterns in model plants like *Arabidopsis thaliana*, further underscores the evolutionary conservation of this family [27,28]. These foundational analyses set the stage for investigating their specific roles in the specialized metabolism of *P. vulgaris*.

Building on this genomic framework, we sought to identify Dof members potentially regulating RA accumulation. Further analysis of cis-regulatory elements revealed that the promoter regions of *P. vulgaris* Dof genes are rich in light-responsive elements (e.g., G-boxes) and hormone-responsive elements (e.g., ABRE, ARE), suggesting their expression may be co-regulated by light signals and hormonal pathways. Transcriptomic data analysis showed distinct tissue-specific expression patterns, with a subset of genes exhibiting high expression in the spike and seed—the primary sites of RA accumulation [29]. This spatial correlation prompted a focused analysis, which identified three candidate genes, *PvDof13*, *PvDof48*, and *PvDof28*, whose expression levels showed statistically significant correlations with RA content across tissues. Notably, *PvDof13* and *PvDof48* expression correlated positively with RA, whereas *PvDof28* showed a strong negative correlation (r = −0.938). This opposing pattern suggests these candidates may function as potential transcriptional activators (*PvDof13/48*) and a repressor (*PvDof28*) in the RA pathway, a functional dichotomy observed within the Dof family in other species [30].

To bridge the observed correlation with a mechanistic hypothesis, we propose that these candidate *PvDofs* may regulate RA biosynthesis by interacting with the promoters of pathway-specific genes. Dof proteins characteristically bind to a core AAAG motif in target gene promoters [17]. Therefore, a critical next step is to investigate whether the promoters of key RA biosynthetic genes, such as rosmarinic acid synthase (*PvRAS*) or hydroxyphenylpyruvate reductase (*PvHPPR*), contain such Dof-binding elements [31]. If present, *PvDof13* and *PvDof48* could act as transcriptional activators, while *PvDof28* might function as a repressor, potentially fine-tuning metabolic flux. We acknowledge that correlation does not establish causation; a gene highly expressed in RA-rich tissues may coincidentally correlate with its content [32]. Thus, our findings highlight strong candidates, not definitive regulators, and their precise roles require functional validation. Furthermore, comparative analysis with transcriptomic studies in related Lamiaceae species (e.g., *Mentha* spp., *Salvia miltiorrhiza*) reveals that Dof genes are often prominently expressed in tissues active in phenolic acid metabolism [24,33], lending evolutionary context and support to the potential relevance of our candidates in specialized metabolism.

As a traditional Chinese medicinal herb, the therapeutic effects of *P. vulgaris* are closely associated with its abundant secondary metabolites, including RA [34]. This study has identified specific Dof transcription factors whose expression patterns correlate with RA accumulation, suggesting their potential regulatory roles. However, the precise molecular mechanisms remain to be elucidated. Future research should employ a combinatorial experimental strategy. First, genetic manipulation (e.g., CRISPR-Cas9, overexpression, or RNAi) in *P. vulgaris* hairy roots or similar systems is necessary to establish causal relationships between the candidate PvDofs and RA biosynthesis [1]. Subsequently, molecular techniques such as chromatin immunoprecipitation (ChIP), electrophoretic mobility shift assays (EMSA), and dual-luciferase reporter assays are essential to directly demonstrate binding of these transcription factors to promoters of RA pathway genes and to quantify their transcriptional effects [35]. This two-tiered approach—from genetic causality to molecular interaction—will be crucial for definitively characterizing the regulatory networks. Additionally, as genomic and multi-omics resources for *P. vulgaris* expand, integrated systems biology analyses will further illuminate the complex regulatory mechanisms governing its valuable secondary metabolism [36].

## 4. Materials and Methods

### 4.1. Plant Materials

The *P. vulgaris* L. plants used in this study were sourced from the medicinal plant garden of Li Shizhen Medical Group in Qichun, Hubei Province, and were identified by Professor Shi Zhaohua (Hubei University of Chinese Medicine) as *P. vulgaris* L. of the Lamiaceae family. The whole-genome data of *P. vulgaris* used was obtained by our research group. The *Arabidopsis thaliana* whole-genome file, annotation file, and protein sequences of its Dof family members were downloaded from the TAIR database (https://www.arabidopsis.org, accessed on 16 June 2025). Tissue Designation: Stem (PVST), Leaf (PVLE), Flower (PVFL), Spike (PVSP), Seed (PVSE). The plant materials used for qRT-PCR were from the same batch as those used for transcriptome sequencing. The genomic data have been deposited in the Genome Warehouse database at the National Center for Bioinformation (Home-Genome Warehouse) under Assembly number GWHHCKR00000000.1 and Project number PRJCA049031.

### 4.2. Experimental Methods

#### 4.2.1. Identification of Dof Gene Family Members in *P. vulgaris*

The Dof conserved domain (PF02701) alignment file was retrieved from the Pfam database (http://pfam.xfam.org, accessed on 16 June 2025). A hidden Markov model (HMM) was built using HMMER (version 3.4) software and used to search against the *P. vulgaris* protein sequences. Simultaneously, the *A. thaliana* Dof protein sequences were used as query sequences for a BLAST (https://blast.ncbi.nlm.nih.gov/Blast.cgi, accessed on 16 June 2025)search against the *P. vulgaris* genome database. The resulting sequences from both methods were merged, and duplicates were removed to obtain candidate *P. vulgaris* Dof transcription factor protein sequences. The Batch CDD search tool in TBtools (version 3.4) software was further employed to analyze whether the candidate *P. vulgaris* Dof transcription factors contained the Dof domain. Sequences lacking the Dof conserved domain were filtered out, yielding the final set of all *P. vulgaris* Dof transcription factor members.

#### 4.2.2. Analysis of Physicochemical Properties and Secondary Structure Prediction of *P. vulgaris* Dof Proteins

The ProtParam tool available on the ExPASy online server (https://web.expasy.org/protparam/, accessed on 16 June 2025) was used to analyze the physicochemical properties of the *P. vulgaris* Dof proteins, including coding sequence length, molecular weight, theoretical isoelectric point (pI), instability index, grand average of hydropathicity (GRAVY), and subcellular localization prediction. Protein secondary structures were predicted online using SOPMA (https://npsa-pbil.ibcp.fr/cgi-bin/npsa_automat.pl?page=npsa_sopma.html, accessed on 16 June 2025).

#### 4.2.3. Chromosomal Localization and Phylogenetic Analysis of the *P. vulgaris* Dof Gene Family

The chromosomal location information for the *P. vulgaris* Dof gene family members was extracted from the *P. vulgaris* genome GFF file using TBtools software and visualized. The amino acid sequences of Dof proteins from *P. vulgaris* and *A. thaliana* were aligned using MEGA (version 11.0) software. A phylogenetic tree was constructed using the Neighbor-Joining (NJ) method with bootstrap replications set to 1000; other parameters were set to default. The resulting tree was visualized and annotated using the iTOL website (https://itol.embl.de, accessed on 17 June 2025).

#### 4.2.4. Domain Analysis of the *P. vulgaris* Dof Gene Family

The conserved motifs in the *P. vulgaris* Dof family protein sequences were analyzed using the online tool MEME (http://meme-suite.org/meme/tools/meme, accessed on 16 June 2025). The site distribution was set to ‘Any number of repetitions’ for a single motif, the motif width was set between 6 and 50 amino acid residues, and the maximum number of motifs to find was set to 10. Gene structure information, including exons and introns, for the *P. vulgaris* Dof gene family members was retrieved from genome annotations and mapped using TBtools software.

#### 4.2.5. Collinearity Analysis of the *P. vulgaris* Dof Gene Family

To further analyze the evolutionary relationships of Dof family members between *P. vulgaris* and *A. thaliana* and within the *P. vulgaris* Dof family itself, interspecific and intraspecific collinearity analyses were performed and visualized using TBtools software.

#### 4.2.6. Cis-Acting Element Analysis in the Promoters of the *P. vulgaris* Dof Gene Family

The 2000 bp genomic sequences upstream of the transcription start sites of the *P. vulgaris* Dof genes were extracted using TBtools software. Cis-acting elements in these promoter regions were predicted online using the PlantCARE database, and the results were visualized.

#### 4.2.7. Protein–Protein Interaction Network Analysis of *P. vulgaris* Dof Family Members

The *P. vulgaris* Dof protein sequences were uploaded to the STRING database website (https://string-db.org, accessed on 16 June 2025). The *A. thaliana* database was selected for sequence alignment to predict interactions. The protein–protein interaction network for the *P. vulgaris* Dof gene family was then assessed and visualized using Cytoscape 3.7.0 software.

#### 4.2.8. Screening of Candidate Dof Genes Potentially Regulating Rosmarinic Acid Biosynthesis in *P. vulgaris*

The correlation between the expression levels of Dof genes and the content of rosmarinic acid from metabolomic data of *P. vulgaris* was analyzed using SPSS software. Based on this analysis, three candidate genes potentially involved in regulating rosmarinic acid biosynthesis in *P. vulgaris* were preliminarily identified.

#### 4.2.9. Expression Pattern Analysis of the *P. vulgaris* Dof Gene Family and Validation by Real-Time Quantitative PCR (qRT-PCR)

Based on previously obtained transcriptome sequencing data from different tissues of *P. vulgaris* (with three biological replicates per tissue), the expression levels of Dof gene family members across five different tissues were extracted. A heatmap was generated using the Heat Map function in TBtools software for visualization. Real-time quantitative PCR (qRT-PCR) was employed to validate the transcript levels of the three screened candidate genes. Total RNA was extracted from different tissues of *P. vulgaris* using the RNAprep Pure Plant Kit (Tiangen, Beijing, China), with RNA quality checked by gel electrophoresis and concentration measured. cDNA was synthesized by reverse transcription using the PrimeScript™ RT Reagent Kit with gDNA Eraser (Takara, Shiga, Japan). The β-actin gene was used as the internal reference gene [37]. Specific qRT-PCR primers for the three genes were designed using Primer 5 software and synthesized by Sangon Biotech (Shanghai, China). The primer sequences are listed in Table 2. The qRT-PCR reaction program was as follows: pre-denaturation at 95 °C for 30 s; 39 cycles of denaturation at 95 °C for 5 s, and annealing/extension at 60 °C for 30 s. Melt curve analysis was performed at 95 °C for 5 s and 60 °C for 30 s. The relative gene expression levels were calculated using the 2^−ΔΔCt^ method [38]. The experiment included three biological replicates to ensure the reliability of the results.

## 5. Conclusions

Based on the whole-genome data of *P. vulgaris*, this study successfully identified 48 Dof transcription factor genes distributed across 14 chromosomes. Bioinformatic analyses revealed that these Dof proteins possess molecular weights ranging from 15,482.44 to 55,875.53 Da, amino acid lengths between 142 and 509, and isoelectric points from 4.84 to 10.2. All proteins were predicted to be localized in the nucleus and classified as hydrophilic. Phylogenetic analysis categorized these Dof genes into four distinct subfamilies, and multiple sequence alignment confirmed that all members contain a conserved C2-C2-type single zinc finger domain. Transcriptome-based expression profiling demonstrated that PvDof genes exhibit clear tissue-specific expression patterns, suggesting potential functional diversification across different tissues of *P. vulgaris*. Protein–protein interaction predictions via the STRING database indicated 242 interactions among 23 Dof proteins. Furthermore, correlation analysis between gene expression levels and rosmarinic acid content identified three key Dof genes that may be involved in the biosynthesis and transcriptional regulation of rosmarinic acid, which was further validated by RT-qPCR. This study provides important theoretical foundations for elucidating the biosynthetic mechanism of rosmarinic acid in *P. vulgaris* and offers potential genetic targets for enhancing its production through genetic engineering.

## Figures and Tables

**Table 1:** Correlation coefficient table. * *p* < 0.05, ** *p* < 0.01 (two-tailed).

		*PvDof48*	*PvDof13*	*PvDof28*	Rosmarinic Acid
*PvDof48*	Pearson Correlation	--			
*PvDof13*	Pearson Correlation	0.833	--		
	Significance (Two-Tailed)	0.080			
*PvDof28*	Pearson Correlation	−0.984 **	−0.833	--	
	Significance (Two-Tailed)	0.002	0.080		
Rosmarinic acid	Pearson Correlation	0.957 *	0.927 *	−0.938 *	--
	Significance (Two-Tailed)	0.011	0.023	0.019	

**Table 2:** qRT-PCR primer sequence information.

Gene Name	Forward Primer Sequence (5′ → 3′)	Reverse Primer Sequence (5′ → 3′)
*PvDof48*	CTATGGTTCCCAAAGACACTCAGA	GATGGGGTTTGGAAGGGTTTAAAG
*PvDof13*	TCAAAGGACGAATTAGGCAAGAGA	GTGTAGCATAGAATGGCATAGGGA
*PvDof28*	TTACAGCCTCTCTCAACCTAGGTA	TTTTGGTGGGTTTAGATCTCGGAT
*β* *-actin*	GACCAGCTCTGCTGTGGAGA	ATGGCTGGAAGAGGACCTCAG

## Data Availability

The original contributions presented in this study are included in the article/Supplementary Material. Further inquiries can be directed to the corresponding author.

## Supplementary Materials

The following supporting information can be downloaded at https://www.mdpi.com/article/10.3390/ijms27031354/s1.
