# Supplementary material for: Genome-Wide Identification and Expression Analysis of the Dof Transcription Factor Family in Prunella vulgaris
Source: Int J Mol Sci. 2026 Jan 29;27(3):1354. doi: 10.3390/ijms27031354 (PMC12898795; doi:10.3390/ijms27031354)
Supplement: Supplementary file 1 [file ijms-27-01354-s001.zip › ijms-4056972-supplementary.pdf]

Table S1 Physicochemical Properties Analysis and Secondary Structure Prediction of *Prunella vulgaris* Dof Protein Family Members

Gene name	Amino acid Length/aa	Relative molecular mass	Isoelectric point	Instability Index	Aliphatic Index	Hydrophilicity	Subcellular localization	Secondary structure/%			
								Alpha-helix	Extension chain	β-sheet	Unregulated curling
PvDof1	287	31898.45	8.49	58.23	57.11	-0.811	Nucleus	20.91	17.07	4.53	57.49
PvDof2	366	39012.06	9.22	62.92	46.67	-0.736	Nucleus	11.2	13.39	6.28	69.13
PvDof3	274	30005.23	8.75	45.41	43.58	-0.824	Nucleus	20.44	12.04	7.3	60.22
PvDof4	346	36227.19	9.38	62.37	49.68	-0.513	Nucleus	13.87	14.16	9.25	62.72
PvDof5	210	21923.44	8.65	30.94	47.57	-0.418	Nucleus	18.57	18.1	7.62	55.71
PvDof7	304	32126.78	8.91	46.9	63.03	-0.384	Nucleus	15.79	16.45	6.91	60.86
PvDof7	272	29946.39	8.75	54.29	49.89	-0.794	Nucleus	12.87	14.34	6.62	66.18
PvDof8	231	24197.46	6.94	52.94	50.3	-0.723	Nucleus	18.61	13.85	9.09	58.44
PvDof9	215	23968.64	8.83	66.51	49.67	-0.731	Nucleus	22.33	13.02	3.72	60.93
PvDof10	235	25024.78	9.25	71.55	41.74	-0.659	Nucleus	20.43	9.36	9.97	60.43
PvDof11	202	22544.53	9.1	58.39	54.01	-0.589	Nucleus	16.34	17.82	4.95	60.89
PvDof12	159	18001.48	9.2	47.16	49.87	-0.857	Nucleus	19.5	11.32	3.14	66.04
PvDof13	509	55875.53	5.42	57.71	42.4	-0.978	Nucleus	15.13	11.59	5.7	67.58
PvDof14	303	32610.33	8.92	57.87	57.13	-0.528	Nucleus	32.01	9.57	7.59	50.83
PvDof15	466	50369.75	6.4	49.66	63.43	-0.653	Nucleus	18.67	12.88	8.58	59.87
PvDof16	249	25957.99	4.61	52.64	52.13	-0.788	Nucleus	16.87	13.25	8.43	61.45
PvDof17	300	32374.13	8.77	52.32	57	-0.476	Nucleus	19.33	14.67	5.67	60.33
PvDof18	288	31210.6	5.9	59.81	62.4	-0.567	Nucle	30.21	12.85	6.6	50.35

		3					us				
PvDof19	321	34964.97	6.58	54.19	54.77	-0.579	Nucleus	16.51	19.31	11.21	52.96
PvDof20	303	34086.5	9.97	52.74	53.43	-0.834	Nucleus	17.49	23.76	6.93	51.82
PvDof21	506	55283.3	5.56	59.52	58.44	-0.767	Nucleus	17.79	11.46	5.53	65.22
PvDof22	231	23996.78	8.94	45.89	52.94	-0.353	Nucleus	8.23	24.68	10.82	56.28
PvDof23	297	32401.19	8.65	43.27	64.81	-0.532	Nucleus	16.16	18.52	6.73	58.59
PvDof24	331	35894.04	6.62	52.26	53.38	-0.621	Nucleus	20.85	19.34	7.55	52.27
PvDof25	306	33418.82	8.9	53.06	46.31	-0.751	Nucleus	18.95	10.78	3.27	66.99
PvDof26	307	33331.75	5.24	56.65	56.32	-0.68	Nucleus	30.29	14.66	4.23	50.81
PvDof27	434	47136.55	5.9	47.93	52.47	-0.773	Nucleus	17.97	13.82	6.68	61.52
PvDof28	246	27174.47	9.51	57.87	58.25	-0.754	Nucleus	14.23	11.79	7.72	66.26
PvDof29	235	24331.96	8.94	40.79	50.34	-0.414	Nucleus	4.68	26.81	8.51	60
PvDof30	481	52366.3	5.62	56.53	56.99	-0.756	Nucleus	21.41	11.85	7.28	59.46
PvDof31	228	24343.48	5.77	59.03	48.82	-0.843	Nucleus	21.49	16.67	7.89	53.95
PvDof32	142	15482.44	8.17	58.59	54.3	-0.66	Nucleus	21.83	16.2	5.63	56.34
PvDof33	266	29127.24	9.46	64.68	40.86	-0.907	Nucleus	10.9	10.15	6.77	72.18
PvDof34	285	30666.69	8.71	62.39	45.23	-0.75	Nucleus	11.58	16.84	6.67	64.91
PvDof35	274	29608.09	6.65	52.7	60.51	-0.578	Nucleus	13.5	18.98	9.12	58.39
PvDof36	319	34655.19	7.61	57.57	49.28	-0.732	Nucleus	28.84	15.67	8.46	47.02
PvDof37	254	27674.9	8.16	55.46	52.76	-0.528	Nucleus	29.53	7.87	6.69	55.91
PvDof38	314	34140.48	7.68	60.42	50.67	-0.77	Nucleus	22.61	17.83	10.19	49.36
PvDof39	231	25641.71	5.07	57.79	49.87	-0.518	Nucleus	28.57	16.88	9.09	45.45
PvDof40	340	36741.2	8.44	57.35	44.26	-0.779	Nucle	15.29	16.76	4.71	63.24

		6					us				
PvDof41	231	25379.31	9.74	62.63	43.16	-0.829	Nucleus	13.85	10.82	6.49	68.83
PvDof42	252	27473.78	7.72	56.22	64.64	-0.564	Nucleus	19.84	17.06	7.14	55.95
PvDof43	265	29594.07	4.47	52.24	47.51	-0.777	Nucleus	22.26	15.47	9.81	52.45
PvDof44	272	28913.23	8.4	51.24	49.49	-0.622	Nucleus	20.96	19.85	12.5	46.69
PvDof45	213	23971.62	8.84	61.03	47.37	-0.758	Nucleus	26.76	9.39	3.76	60.09
PvDof46	278	29498.77	9.67	72.03	42.66	-0.661	Nucleus	23.74	11.51	10.07	54.68
PvDof47	161	18017.48	9.2	45.44	45.59	-0.815	Nucleus	11.18	16.15	8.07	64.6
PvDof48	487	53171.49	5.02	57.65	44.93	-0.956	Nucleus	16.84	10.88	6.16	66.12

Table S2 Conserved Motifs of Dof Transcription Factors in *Prunella vulgaris*

	Domain Sequence	Motif Visualization
1	CPRCBSTNTKFCYYNNYSLSQPRHFCKSCRRYWTKGGTLRNVPVGGGCRK	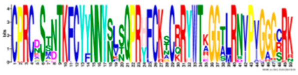
2	EKNLWVPKTLRIDDPGEAAKSSIWATLGIKNE	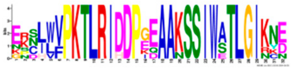
3	DPKIKLFGKTIELPE	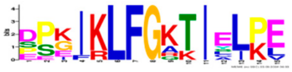
4	DEKKHVLETSTVLQANPAALSRSLNFHE	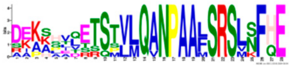
5	ALNPNGTVJTFGSDSPLCESMVSVLNIVDKTN	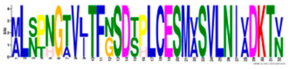
6	MERKLRPZKEQALK	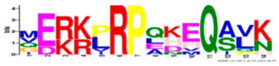
7	DQNETSNSQDKTLKKPDKILP	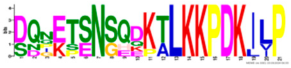
8	VPCYPGGPWPYPWNPMQWNPAISPPNFPMPFY	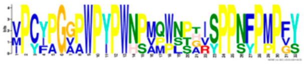
9	SPTLGKHSRDDNILKPANTEE	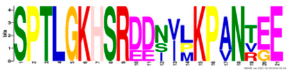
10	HDLNLAYNPSSHYNAMSEFAAMQHHSMGNPNSDNGPQRPHHHNLPLMELL	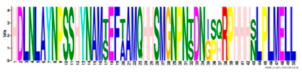

Supplementary Table S3: Abbreviations for cis-acting elements used in Figure 7

No.	Abbreviation	Full Name
1	**LRE**	light responsive element
2	**LR-cis**	cis-acting regulatory element involved in light responsiveness
3	**LRM**	part of a conserved DNA module involved in light responsiveness
4	**LRE-part**	part of a light responsive element
5	**AuxRE**	auxin-responsive element
6	**CCR**	cis-acting element involved in cell cycle regulation
7	**SARE**	cis-acting element involved in salicylic acid responsiveness
8	**ARE (Anaerobic)**	cis-acting regulatory element essential for the anaerobic induction
9	**DSRE**	cis-acting element involved in defense and stress responsiveness
10	**LTRE**	cis-acting element involved in low-temperature responsiveness
11	**LRM-part**	part of a module for light response
12	**SRE**	cis-acting regulatory element involved in seed-specific regulation
13	**MeJA-RE**	cis-acting regulatory element involved in the MeJA-responsiveness
14	**ABRE**	cis-acting element involved in the abscisic acid responsiveness
15	**CRE**	cis-acting regulatory element
16	**PBS**	protein binding site
17	**GARE**	cis-acting element involved in gibberellin-responsiveness
18	**ZMRE**	cis-acting regulatory element involved in zein metabolism regulation

No.	Abbreviation	Full Name
19	**PMD**	element involved in differentiation of the palisade mesophyll cells
20	**ASE**	enhancer-like element involved in anoxic specific inducibility
21	**LR-cis2**	cis-acting element involved in light responsiveness
22	**MBS-drought**	MYB binding site involved in drought-inducibility
23	**ATBP-1 BS**	binding site of AT-rich DNA binding protein (ATBP-1)
24	**Amy-con**	sequence conserved in alpha-amylase promoters
25	**AuxRE-cis**	cis-acting regulatory element involved in auxin responsiveness
26	**MRE**	cis-acting regulatory element related to meristem expression
27	**CCE**	cis-acting regulatory element involved in circadian control
28	**MYBHv1 BS**	MYBHv1 binding site
29	**AuxRE-part**	part of an auxin-responsive element
30	**gapA-LR**	part of gapA in (gapA-CMA1) involved with light responsiveness
31	**LRM-part2**	part of a light responsive module
32	**MBS-flavonoid**	MYB binding site involved in flavonoid biosynthetic genes regulation
33	**MBS-light**	MYB binding site involved in light responsiveness
34	**MSA**	cis-acting regulatory element related to meristem specific activation
35	**END**	cis-regulatory element involved in endosperm expression
36	**MEA (2x)**	element for maximal elicitor-mediated activation (2 copies)
37	**GARE-alt**	gibberellin-responsive element
38	**END-neg**	involved in endosperm-specific negative expression
